# Polyoma BK Virus: An Oncogenic Virus?

**DOI:** 10.1155/2013/858139

**Published:** 2013-07-07

**Authors:** Syed Hassan, Zaid Alirhayim, Syed Ahmed, Syed Amer

**Affiliations:** ^1^Department of Internal Medicine, Henry Ford Hospital, 2799 West Boulevard, Detroit, MI 48202, USA; ^2^Department of Medicine, Deccan College of Medical Sciences, Hyderabad 500058, India; ^3^Department of Internal Medicine, Brookdale University Hospital and Medical Centre, Brooklyn, NY 11212, USA

## Abstract

We report a case of a 65-year-old gentleman with a history of end stage renal disease who underwent a successful cadaveric donor kidney transplant four years ago. He subsequently developed BK virus nephropathy related to chronic immunosuppressant therapy. Three years later, misfortune struck again, and he developed adenocarcinoma of the bladder.

## 1. Introduction

BK virus is a member of the polyomavirus family. In immunocompetent individuals it is innocuous, often remaining latent in renal and urothelial cells [1]. Once immunity is compromised, such as in the renal transplant recipient, BK virus may result in graft dysfunction and even loss of the entire graft, an entity known as BK virus associated nephropathy (BKVAN) [2]. The existing literature suggests that persistently elevated levels of BK virus within transitional urothelial epithelium are associated with tumour formation and malignancy [3]. In this particular case, we attempt to highlight the potential pathologic role of BK virus in bladder cancer and in the renal transplant setting. 

## 2. Case

A 65-year-old man received a cadaveric kidney transplant after developing end stage renal disease. He had a longstanding history of hypertension and was receiving lithium therapy for many years for schizophrenia. Postoperatively, he established excellent graft function and was able to urinate normally. For long term immunosuppression, he was placed on oral prednisone, tacrolimus, and mycophenolate. In the months that followed, he continued to attend the transplant clinic on a regular basis. Six months postoperatively, he was noted to have elevated BUN and creatinine levels of 38 mg/dL and 1.86 mg/dL, respectively, from a previously normal baseline. Tacrolimus levels were checked to exclude its toxicity. A renal ultrasound was performed but was found to be negative for any leak or obstruction. BK levels were checked, and he was found to have a viral load greater than 400,000 copies/mL on two separate occasions. Doses of his immunosuppressant medications were immediately reduced. Over the next four years, he continued to undergo close surveillance with regular monitoring of serum creatinine and BK virus DNA levels. In spite of our best efforts, he eventually developed renal allograft failure. Histopathological specimens were sent. Immunohistochemical staining using the peroxidase test for SV-40 antigen was performed, and the diagnosis of renal failure secondary to BKVAN was confirmed. The patient was initiated on haemodialysis and placed on the transplant list once more. He continued to see his primary care physician on a routine basis. During one such visit, he complained of hematuria. Imaging studies of the abdomen including the transplanted kidney were unremarkable. A urinalysis showed 40–60 RBCs per HPF, mild proteinuria, but was otherwise negative. Retrograde pyelography was performed and revealed a medium sized sessile tumour. Cystoscopy showed it to be a tumour in the posterior wall of the bladder, and a transurethral resection of the tumour was done. Pathology specimens confirmed a high grade, adenocarcinoma in situ of the bladder, not invading into the muscle wall. CT imaging studies of the abdomen and pelvis did not show any metastatic disease. In order to exclude a gastrointestinal stromal tumour (GIST), esophagogastroduodenoscopy and colonoscopy were performed and found to be negative. Follow-up imaging and cystoscopy six months later did not show residual tumour burden. The patient continues to do relatively well and is listed for a second kidney transplant. 

## 3. Discussion

BK virus is associated with several cancers in transplant recipients, particularly kidney transplant patients. Since it was first isolated in 1971, several hypothesis have emerged to explain its potential oncogenic role [4]. One theory is that the BK virus strongly expresses a viral antigen, SV-40 T, which acts to suppress tumour suppressor proteins of the pRB family. This causes progression into the G2/S phase of the cell cycle and further cell proliferation. This may be further enhanced by binding of the T antigen to the p53 tumour suppressor gene with resultant inhibition of apoptosis and DNA repair [5]. In transgenic mouse models, T antigen has been shown to deactivate p53 and pRB producing renal urothelial neoplasms analogous to those seen in humans [6]. McCabe et al. proposed an alternate theory suggesting that T antigen activates the DNA methyl transferase-1 gene resulting in hyper methylation of the tumour suppressor genes and therefore tumour genesis [7]. This is concordant with studies that utilized immunohistochemical methods to demonstrate accumulation of p53in BK virus infected urothelial cells [3].

Several case studies have incriminated BK virus in bladder and prostrate carcinomas, Kaposi sarcoma, and bone and brain tumours [8–11]. Bladder carcinoma has even been reported in a patient who had undergone simultaneous kidney and pancreas transplant [12]. In this particular patient, bladder carcinoma developed in a patient with a known history of BK viremia. This case is an addition to the accumulating body of evidence implicating BK virus in the development of malignancies.

## References

[1] Drachenberg CB, Hirsch HH, Ramos E, Papadimitriou JC (2005). Polyomavirus disease in renal transplantation: review of pathological findings and diagnostic methods. *Human Pathology*.

[2] Thamboo TP, Jeffery KJM, Friend PJ, Turner GDH, Roberts ISD (2007). Urine cytology screening for polyoma virus infection following renal transplantation: the Oxford experience. *Journal of Clinical Pathology*.

[3] Roberts ISD, Besarani D, Mason P, Turner G, Friend PJ, Newton R (2008). Polyoma virus infection and urothelial carcinoma of the bladder following renal transplantation. *British Journal of Cancer*.

[4] Gardner SD, Field AM, Coleman DV, Hulme B (1971). New human papovavirus (B.K.) isolated from urine after renal transplantation. *Lancet*.

[5] Tognon M, Corallini A, Martini F, Negrini M, Barbanti-Brodano G (2003). Oncogenic transformation by BK virus and association with human tumors. *Oncogene*.

[6] Garcia-España A, Salazar E, Sun TT, Wu XR, Pellicer A (2005). Differential expression of cell cycle regulators in phenotypic variants of transgenically induced bladder tumors: implications for tumor behavior. *Cancer Research*.

[7] McCabe MT, Low JA, Imperiale MJ, Day ML (2006). Human polyomavirus BKV transcriptionally activates DNA methyltransferase 1 through the pRb/E2F pathway. *Oncogene*.

[8] Das D, Wojno K, Imperiale MJ (2008). BK virus as a cofactor in the etiology of prostate cancer in its early stages. *Journal of Virology*.

[9] Monini P, Rotola A, de Lellis L (1996). Latent BK virus infection and Kaposi's sarcoma pathogenesis. *International Journal of Cancer*.

[10] Weinreb DB, Desman GT, Amolat-Apiado MJM, Burstein DE, Godbold JH, Johnson EM (2006). Polyoma virus infection is a prominent risk factor for bladder carcinoma in immunocompetent individuals. *Diagnostic Cytopathology*.

[11] Negrini M, Rimessi P, Mantovani C (1990). Characterization of BK virus variants rescued from human tumours and tumour cell lines. *Journal of General Virology*.

[12] Geetha D, Tong BC, Racusen L, Markowitz JS, Westra WH (2002). Bladder carcinoma in a transplant recipient: evidence to implicate the BK human polyomavirus as a causal transforming agent. *Transplantation*.

